# Migraine headaches among university students using id migraine test as a screening tool

**DOI:** 10.1186/1471-2377-11-103

**Published:** 2011-08-19

**Authors:** Serdar Oztora, Osman Korkmaz, Nezih Dagdeviren, Yahya Celik, Ayse Caylan, Mehmet S Top, Talip Asil

**Affiliations:** 1 Trakya University, Faculty of Medicine, Department of Family Medicine, Edirne, Turkey; 2 Trakya University, Faculty of Medicine, Department of Neurology, Edirne, Turkey; 3 Ministry of Health, Edirne State Hospital, Department of Psychiatry, Edirne, Turkey

## Abstract

**Background:**

Migraine is a significant health problem, especially for the young people, due to its frequency and accompanying morbidity, causing disability and loss of performance. In this study, our aim was to determine the prevalence of migraine headaches among university students in Edirne, a Turkish city.

**Methods:**

In this cross-sectional and descriptive study, study population was composed of students registered to Trakya University in the academic year of 2008-2009. Out of these, 3694 of them accepted to participate. Participants who had two or more headaches in the last 3 months formed the headache group. Afterwards, two preliminary questions were applied to the headache group and participants with at least one affirmative response were asked to perform the validated ID-Migraine™ test.

**Results:**

The mean age of 3694 students participated in the study was 19.23 ± 1.84 (17-39 years), with adolescents:adult ratio being 2.5:1. 1613 students (43.7%) did have at least two headaches in the last three months. Migraine-type headache was detected in 266 subjects (7.2%) based on the ID-Migraine™ test. Of the migraine group, 72 were male (27.1%) and 194 were female (72.9%). There was no significant difference in migraine prevalence between adolescent and adult age groups.

**Conclusions:**

With a prevalence similar to adults, primary care physicians should be aware of the probability of migraine headaches in university students in order to maintain a successful school performance.

## Background

Migraine is an important health problem that affects more than 10% of the general population [1-3]. There are various prevalence rates of migraine type headaches. The variety of prevalence in different studies depends on the method used to diagnose migraine-type headaches. In the first nationwide cross-sectional study, the headache profile of Turkey has been revealed and the life-time migraine prevalence of migraine type headaches was reported to be 16%, which was 10.9% in men and 21.8% in women [4]. There are few studies focused on the prevalence of migraine in university students in Turkey [5-7]. Due to its negative effects on quality of life, it becomes especially considerable among university students, who require constant concentration and performance [8,9]. Headaches have a profound impact on school performance among university students. This impact is more evident among migrainous students than students with episodic tension-type headaches (ETTH) with a 62.7% decrease in capacity versus 24.4% respectively. Moreover, students with migraine type headaches missed more school than students with ETTH [8]. These findings reveal the importance of migraine headaches in university students. To be able to determine the prevalence, useful, reliable and validated screening tests must be applied to such large populations. Identity Migraine (ID Migraine™) test is a proper and useful screening tool, developed and validated by Lipton et al. [10,11], which can be applied quickly to large numbers of populations. Sensitivity, specificity and positive predictive value of this test in primary care have been defined as 81%, 75% and 93%, respectively [11]. The validation of ID Migraine™ test in Turkish has been made by Karli with a sensitivity of 91.8%, specificity of 63.4%, positive predictive value of 71.9% and a negative predictive value of 88.4% [12]. As World Health Organization (WHO) defines adolescence as young people between the ages of 10 and 19 years, university population consists of both adolescents and adults. The test is also validated to be used among adolescent students with a sensitivity of 62.1% and specificity of 71.1% [13]. In this study, we aimed to determine the prevalence of migraine headaches among university students using the quick screening tool ID Migraine™ test.

## Methods

In 2008-2009 academic year, 4645 students registered to different faculties of Trakya University, Edirne, has been reached and asked to participate in the study. Of the registrants, 3732 students agreed to participate and filled out the questionnaire. The study was conducted in accordance with the Declaration of Helsinki and Good Clinical Practice Guidelines and approved by the institutional review board. Participants were informed and their verbal consent was obtained. After the data collection, 3694 participants were included in the study. The rest were excluded due to incomplete questionnaire or inconsistent responses.

**Figure 1:**
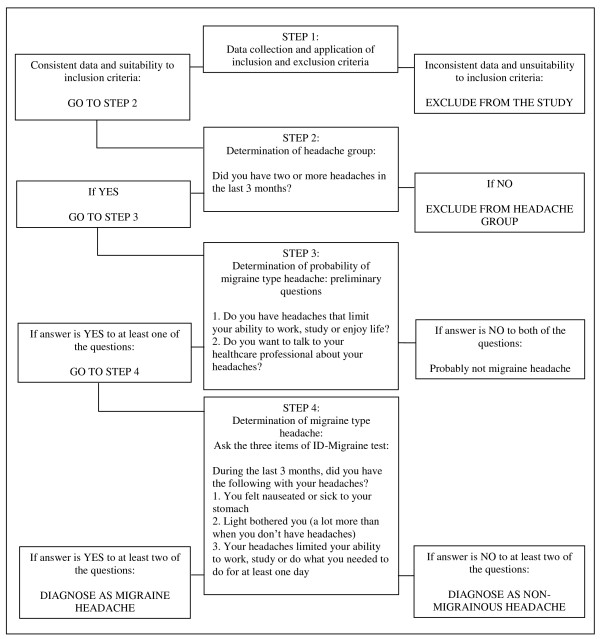
**Flow-chart of the study process**.

A stepwise evaluation is used to determine the prevalence of migraine type headache. Students who replied "yes" to the first question "Did you have two or more headaches in the last 3 months?" were considered as the subjects with headache and asked the preliminary questions. Participants giving one positive response out of two were considered as the subjects who are likely to have migraine type headaches and asked the 3- item ID Migraine™ test. Subjects with two positive responses out of those three were considered having migraine headache (Figure 1).

For the statistical analysis of the cross-sectional, descriptive study, Statistical Package for the Social Sciences (SPSS) for Windows was used.

## Results

**Figure 2:**
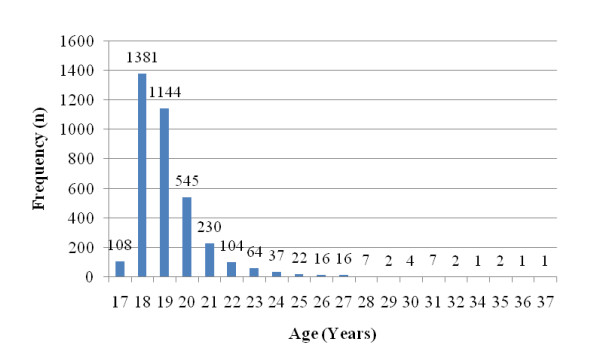
**Participants according to age groups**.

At the end of the study, data collected from 3694 participants were analyzed. Of the participants, 50.1% were male (n = 1850) and 49.9% female (n = 1844). The mean age of the participants was 19.23 ± 1.84, ranging from 17 to 37 years of age (Figure 2). 1613 students (43.7%) who replied "yes" to the question "Did you have two or more headaches in the last 3 months?" formed the headache group. Of the headache group, 43.8% were male (n = 706) and 56.2% female (n = 907). Female participants had significantly higher headache rates than male ones (Pearson χ^2 ^= 45.628, p < 0.001). Details are shown in Table 1.

**Table 1:** Headache group according to gender

Did you have two or more headaches in the last 3 months?			
	Yes	No	Total
Male	706 (19.1%)	1144 (31.0%)	1850 (50.1%)
Female	907 (24.6%)	937 (25.3%)	1844 (49.9%)

Total	1613 (43.7%)	2081 (56.3%)	3694 (100.0%)

Pearson χ^2 ^= 45.628, p < 0.001.

As preliminary questions were analyzed, 815 participants of the headache group (50.5%) had at least one positive response. 463 participants (28.7%) had "a desire to talk to a healthcare professional about this headaches", and 633 participants expressed that "his/her ability to work, study or enjoy life was limited" (39.2%).

This group of participants was further evaluated in terms of migraine type headaches with application of the 3-item ID Migraine™ test. Of this group, 32.6% (n = 266) gave at least two positive responses and had positive ID Migraine™ tests. The prevalence of migraine among all participants according to ID Migraine™ test was 7.2%. Of the migraine group, 72 were male (27.1%) and 194 were female (72.9%). The rate of migraine in female participants was found to be significantly higher than male ones (Pearson χ^2 ^= 60.725, p < 0.001). The mean age of the participants with migraine was 19.35 ± 1.84, ranging from 17 to 29 years of age. There was no statistical significance between the ages of participants and migraine prevalence (Pearson χ^2 ^= 16.958, p = 0.593). Of the participants, 71.3% (n = 2633) were in the adolescent age, whereas 28.7% (n = 1061) were in the adult group, with a ratio of 2.5:1. In comparison, there was no significant difference between adolescent and adult age groups concerning the migraine rates (7.0% and 7.8%, respectively) (Table 2). In addition, Table 3 shows the prevalence rates according to gender and age.

**Table 2:** Comparison between adolescent and adult age groups

	Id-Migraine™ results		
	Migrainous	Non-Migrainous	Total
Adolescent (≤19 years of age)	183 (7.0%)	2450 (93.0%)	2633 (100%)

Adult (>19 years of age)	83 (7.8%)	978 (92.2%)	1061 (100%)

Total	266	3428	3694

Pearson χ^2 ^= 0.862, p = 0.353.

**Table 3:** Migraine prevalence according to gender and age

		Positive ID-Migraine test		
		n	%	p
Gender	Male	72	3.9	Pearson χ^2^=
	Female	194	10.5	60.725,
	Total	266	7.2	p < 0.001

Age	17-19 yrs.	183	7.0	Pearson χ^2^=
	20-24 yrs.	75	7.7	0.862,
	25-37 yrs.	8	9.9	p = 0.493
	Total	266	7.2	

In the migraine group (n = 266), 3 item ID Migraine™ test showed that 75.2% of the participants (n = 200) "felt nauseated or sick to the stomach", 86.8% of the participants (n = 231) were "bothered by light (a lot more than when they don't have headaches)", and 72.9% (n = 194) expressed that "their headaches limited their ability to work, study or do what they needed to do for at least one day".

## Discussion

This study has been conducted among adolescents and adults registered to different faculties of Trakya University, Edirne, Turkey. ID Migraine™ test has been used for screening migraine type headaches. ID Migraine™ test showed that, 7.2% (n = 266) of the 3694 students had migraine type headache.

The prevalence of migraine is reported to be the highest between ages 30-39 [1,4]. However it is also frequent among adolescents and university students [13]. Age did not have a relationship with the prevalence of migraine in our study. There was also no significant difference between adolescent and adult populations. Thus, the young people, often thought of as a healthy group, should not be parted from adults in terms of migraine type headaches. There are few studies focusing on migraine among university students in Turkey showing differences in prevalence. Demirkiran reported a prevalence of 12.4% among 1029 students, whereas Bicakci reported 21.9% among 1256 students [5,7]. Kurt found that 17.9% of 2023 students had migraine-type headache [14]. Several studies throughout the world show different results. The prevalence of migraine among university students is reported to be 8.9% in a Croatian study among 314 students, 7.0% in a Norwegian study among 5847 students, and 6.4% in a Nigerian study among 376 students [15-17]. We found the prevalence of migraine headaches relatively lower than Turkish, but similar to international studies. Our relatively larger study population could have an effect on the results. As the screening test is based on the presence of headache in the last three months, the time of the study can also be a limitation factor, which differs with various studies. A study conducted in more stressful periods like midterms or final examinations would have revealed a higher prevalence of migraine headaches. Also, the methods used to determine the prevalence of migraine type headaches can significantly affect the prevalence rates explaining the differences observed in the epidemiological studies. Fewer studies are present using the validated ID Migraine™ test with only one similar study population, which is performed in southern Turkey by Bicakci et al. [7,10-13].

The female/male ratio was 2.7:1 (194/72) in our study, whereas in two other cities in Turkey, it was 3.4:1 in Afyon and 1.2:1 in Adana [5,7]. It is compatible with the literature, as it is known that migraine is more common in female than male gender [18,19].

The importance of migraine headaches among university students lies within its disability potential [20,21]. ID Migraine™ test items investigate the major aspects of migraine type headaches, which are nausea, photophobia and disability. Of the participants, 72.9% (n = 194) experienced limitation of ability to work or study for at least one day, whereas 75.2% of the participants (n = 200) had nausea, 86.8% of the participants (n = 231) had photophobia. Along with the discomfort caused by the headache, these results show a significant limitation of daily routine. As university curriculum requires constant concentration and hard work, even a one-day absence of the student can affect his/her school success. Combined with the fact that there are also 1347 students (36.4%) having non-migraine headaches, evaluating and managing headaches among university students become especially important.

Although there is a previous study among university students using ID-Migraine test in a different geographical region of Turkey, we think that our research is useful as it confirms the opportunity to evaluate migraine prevalence in a population where the disease could exert a relevant negative impact [7].

## Conclusions

Although migraine is thought to be an adult condition, it is common among adolescents as well. It is still an underestimated condition, even in neurology practice [22,23]. Thus it can easily be missed in adolescents, as well as the adults. In order to maintain a successful school performance, primary care physicians should be aware of the probability of migraine headaches [24]. ID Migraine™ test, being a useful, valid and quick screening tool, will help primary care physicians in order to diagnose migraine in adolescents as well.

## Competing interests

The authors declare that they have no competing interests.

## Authors' contributions

SO participated in the design of the study, performed the statistical analysis and drafted the manuscript. OK carried out the data collection and participated in the design of the study. ND participated in the design and coordination of the study, helped to perform the statistical analysis and draft the manuscript. YC and AC participated in the design and coordination of the study and helped to draft the manuscript. MST and TA participated in its design and coordination. All authors read and approved the final manuscript.

## Pre-publication history

The pre-publication history for this paper can be accessed here:

http://www.biomedcentral.com/1471-2377/11/103/prepub
